# Low-Temperature, Dry Transfer-Printing of a Patterned Graphene Monolayer

**DOI:** 10.1038/srep17877

**Published:** 2015-12-09

**Authors:** Sugkyun Cha, Minjeong Cha, Seojun Lee, Jin Hyoun Kang, Changsoon Kim

**Affiliations:** 1 Program in Nano Science and Technology, Graduate School of Convergence Science and Technology, Seoul National University, Seoul 151-742, Republic of Korea; 2 Department of Chemistry, Seoul National University, Seoul 151-747, Republic of Korea; 3 Advanced Institutes of Convergence Technology, Suwon, Gyeonggi 443-270, Republic of Korea

## Abstract

Graphene has recently attracted much interest as a material for flexible, transparent electrodes or active layers in electronic and photonic devices. However, realization of such graphene-based devices is limited due to difficulties in obtaining patterned graphene monolayers on top of materials that are degraded when exposed to a high-temperature or wet process. We demonstrate a low-temperature, dry process capable of transfer-printing a patterned graphene monolayer grown on Cu foil onto a target substrate using an elastomeric stamp. A challenge in realizing this is to obtain a high-quality graphene layer on a hydrophobic stamp made of poly(dimethylsiloxane), which is overcome by introducing two crucial modifications to the conventional wet-transfer method – the use of a support layer composed of Au and the decrease in surface tension of the liquid bath. Using this technique, patterns of a graphene monolayer were transfer-printed on poly(3,4-ethylenedioxythiophene):polystyrene sulfonate and MoO_3_, both of which are easily degraded when exposed to an aqueous or aggressive patterning process. We discuss the range of application of this technique, which is currently limited by oligomer contaminants, and possible means to expand it by eliminating the contamination problem.

Graphene, a one-atom-thick layer of carbon atoms arranged in a hexagonal lattice, has outstanding electrical^1^,^2^ and mechanical^3^,^4^ properties, as well as high optical transmittance^5^. For this reason, many electronic and photonic devices employing graphene, as either an active layer or a transparent electrode, have been demonstrated, such as light-emitting diodes (LEDs)^6^,^7^, solar cells^8^,^9^, field-effect transistors^10^, photodetectors^11^, touch screens^12^, terahertz wave modulators^13^,^14^,^15^, and Schottky junction devices^16^,^17^. In many such demonstrations, a graphene layer has been deposited by transferring it onto a device substrate following the conventional wet-transfer method, where a graphene–polymer bilayer floating on a water bath is scooped by the substrate^18^. And when the patterning of graphene layers is required, it has mostly been performed after graphene transfer, typically using photolithography followed by reactive-ion etch (RIE)^19^,^20^. However, this method of obtaining patterned graphene layers – the wet-transfer and subsequent patterning process – has only a limited range of applications, where graphene layers must be deposited and patterned, when necessary, prior to deposition of any material that is too fragile to withstand a wet, high-temperature, or plasma process. Notable, practically important examples of such materials are organic semiconductors^21^ and organometal trihalide perovskite compounds^22^.

Attention, therefore, has been focused on development of dry-transfer techniques^23^. For example, a graphene layer grown on a Cu layer on a donor substrate can be directly transferred onto a target substrate, by delaminating the graphene–Cu interface when the target substrate in contact with the graphene layer is peeled off from the donor substrate^24^. However, for selective delamination, the target substrate needs to be coated with an epoxy adhesion layer, which makes this technique unsuitable for high-performance electronic devices: for example, it cannot be applied to fabrication of an LED with a top graphene electrode, since the adhesion layer in this case would be placed in the device interior, just beneath the graphene electrode, impeding efficient charge injection. Another approach is to transfer-print a graphene layer coated with a ‘self-release’ layer from an elastomeric stamp onto a target substrate^25^, where reliable transfer is achieved by choosing an appropriate self-release layer that assures the selective delamination at the interface between that and the elastomer. Although the transfer process itself is dry, removing the self-release layer transferred along with the graphene is typically achieved with an organic solvent, ultimately limiting applications of this method. Jung *et al*. demonstrated a technique capable of transferring graphene monolayers without an adhesion or a self-release layer^26^. In this mechano-electro-thermal process, complete transfer, instead, requires application of high temperature (≥160 °C) and voltage (≥600 V) while a graphene layer grown on Cu foil is pressed onto a target substrate.

Here, we demonstrate a low-temperature, dry transfer process capable of transfer-printing a patterned graphene monolayer onto a target substrate that can be damaged or degraded by a wet, plasma or high-temperature process. In this process, a graphene monolayer on Cu foil, which is grown by chemical vapor deposition (CVD) and then patterned using a conventional lithographic process, is transferred onto a stamp made of poly(dimethylsiloxane) (PDMS), and subsequently transfer-printed from the stamp onto the target substrate. The graphene transfer from Cu foil to PDMS is achieved using the conventional wet-transfer process^18^, with the following two modifications: the use of Au, instead of poly(methyl methacrylate) (PMMA), as a material for the support layer, and the decrease in surface tension of the liquid bath using a water-ethanol mixture. These modifications are critical in preventing defect formation in a graphene monolayer during its transfer onto a PDMS stamp, thereby leading to a minimum sheet resistance of 573 Ω/sq for a graphene monolayer transfer-printed onto a glass substrate. Furthermore, we demonstrate transfer-printing of patterned graphene monolayers on poly(3,4-ethylenedioxythiophene):polystyrene sulfonate (PEDOT:PSS) and MoO_3_, which are representative examples of organic electronic materials and practically important metal oxides^27^, respectively, that are usually damaged or degraded when exposed to aqueous or aggressive patterning processes. The morphological and elemental characterizations of the surfaces of transfer-printed graphene show the existence of contaminants that are likely to be siloxane oligomers transferred from the PDMS stamp. We discuss the current range of application of this technique and possible means to expand it by eliminating the contamination problem.

## Transfer-Printing of a Patterned Graphene Layer

**Figure 1:**
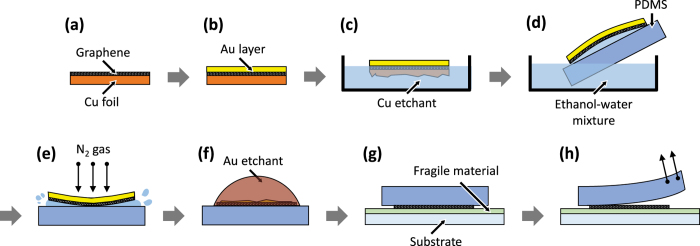
Transfer-printing of a CVD-grown graphene monolayer. The process begins with a graphene monolayer grown on Cu foil (**a**). After depositing a support layer composed of Au (**b**), the Cu layer is etched by floating the sample on a Cu etchant bath (**c**). After transferring the resulting graphene–Au bilayer to a bath comprising a mixture of water and ethanol, the bilayer was scooped up with a PDMS stamp (**d**). Next, the sample is blow-dried using a N_2_ gun (**e**), followed by heat treatment on a hot plate. Removing the Au layer (**f**) completes the fabrication of the stamp coated with the graphene monolayer, which is then gently pressed onto a target substrate coated with a material that can be easily damaged by a wet process (**g**). The graphene monolayer is transferred onto the substrate as the stamp is peeled off from the substrate (**h**). Between (**g**) and (**h**), the stamp and substrate are kept in conformal contact for 1 h, followed by heat treatment on a hot plate.

To transfer a graphene monolayer onto a target substrate that can be damaged or degraded by a wet or high-temperature process (Fig. 1), we first transfer a CVD-grown graphene onto a PDMS stamp following the conventional wet-transfer method (a to f): by scooping up, with the PDMS stamp, a graphene–support bilayer floating on liquid. After the support layer is removed by chemical etching, the graphene is transfer-printed on a target substrate (g to h). The first part of this process (a to f), although seemingly similar to the conventional wet-transfer technique^18^, has two distinct features, which are crucial to obtain a high-quality graphene monolayer on a target substrate.

First, as a support layer material, we use thermally deposited Au, instead of PMMA, which is most widely used for this purpose in the wet-transfer method^28^. PDMS, the material chosen for a stamp owing to its mechanical and chemical properties suitable for various transfer-printing techniques^29^, swells when immersed in an organic solvent^30^ that can dissolve the PMMA support layer, such as acetone and chloroform. When this occurs, the graphene monolayer cracks, creating a large number of defects (Supplementary Fig. S1). On the contrary, the use of a Au support layer allows one to obtain a high-quality graphene monolayer on PDMS, since Au can be removed using an aqueous etchant, which does not swell PDMS.

Second, for the liquid on which the graphene–Au bilayer floats and from which it is scooped with a PDMS stamp [Fig. 1(d)], we use an ethanol–water mixture, instead of water commonly used in the conventional wet-transfer technique. This is to decrease the surface tension of the liquid. In the conventional case, after the graphene–support bilayer is scooped with a hydrophilic substrate [as in Fig. 1(d)], a thin layer of water is present throughout the graphene–substrate interface, providing sufficient lubrication at that interface. As a result, when the sample is blow-dried using a N_2_ gun, the graphene and substrate form a conformal contact without wrinkles throughout the substrate, as the water is laterally displaced [Supplementary Fig. S2(a)]. Since the surface of a PDMS stamp is hydrophobic, which is favorable for reliable transfer of a graphene monolayer onto a target substrate via stamping (g to h in Fig. 1), the use of water bath in Fig. 1(d) leads to a discontinuous lubrication layer between the bilayer and substrate, as schematically shown in Supplementary Fig. S2(b). Therefore, blow-drying in this case results into bursting of trapped water droplets, tearing the graphene monolayer. This can be effectively prevented by using an ethanol–water mixture as the liquid bath, which sufficiently wets the PDMS surface to provide a continuous lubrication layer [Supplementary Fig. S2(c)].

When patterning of graphene is required, a conventional patterning process, such as O_2_ RIE of graphene using photoresist patterned by photolithography as an etch mask^19^,^20^, is performed before Step (b) in Fig. 1. Then, performing the remaining processes [Step (b) to (h)], one can obtain a patterned graphene monolayer on a target substrate. This pre-transfer patterning of graphene allows one to avoid possible damage to the fragile material that is likely to occur, when a process such as photolithography^19^,^20^, RIE^19^,^20^, or laser ablation^31^ is performed after the graphene is transferred to the target substrate.

## Results and Discussion

**Figure 2:**
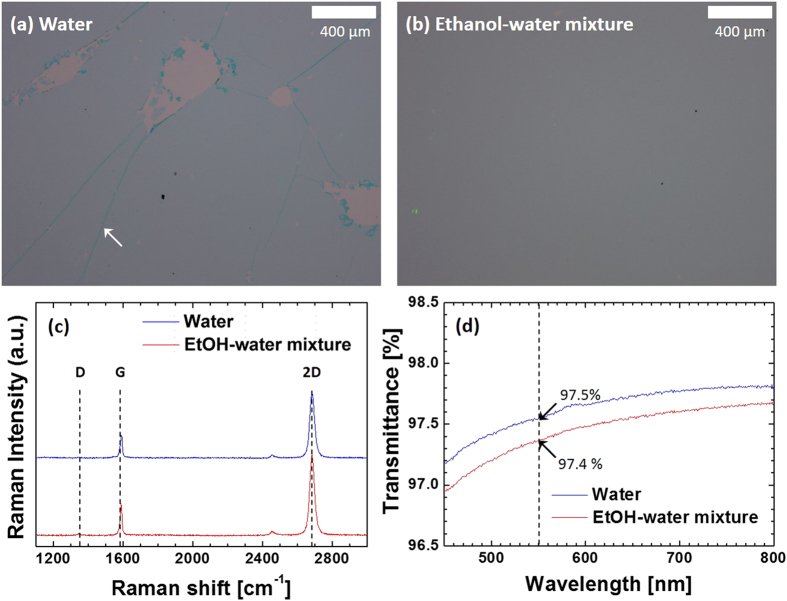
Effect of surface tension on the quality of transfer-printed graphene monolayers. (**a**,**b**) Optical microscope images of graphene monolayers transfer-printed on Si substrates pre-coated with SiO_2_. The bath used in step (d) in Fig. 1 is composed of water in (**a**) and a water–ethanol mixture in (**b**). Along a line indicated by the arrow in (**a**), the graphene monolayer is folded, which arises from a corresponding wrinkle in the graphene–Au bilayer formed due to insufficient wetting of the PDMS surface by the bilayer. (**c**) Raman spectra of the graphene monolayers on the Si/SiO_2_ substrates. (**d**) Optical transmittance spectra of the graphene monolayers transferred on glass substrates. Each curve was obtained by averaging over five samples.

To show that the surface tension of a liquid used in Step (d) in Fig. 1 is a critical factor determining the quality of transfer-printed graphene, we transfer-printed a graphene monolayer on a Si substrate coated with a 285-nm-thick SiO_2_ layer following a process described in Fig. 1, while varying the liquid bath: in one set of experiments, we used water, and in the other, a water–ethanol mixture (30% water and 70% ethanol by volume). When water bath was used, although the entire graphene sheet (1.3 cm by 1.3 cm) was seemingly well-transferred, a closer observation revealed that there are randomly distributed irregular-shaped holes where graphene is absent, as shown in Fig. 2(a). The density of these defects is approximately 10 cm^–2^, which was obtained by counting the number of defects distributed over the whole sample area using an optical microscope. When the PDMS stamp was observed by an optical microscope after Step (f) in Fig. 1, it was found that similar defects, albeit smaller in size, were present (Supplementary Fig. S3), indicating that the defects are formed while transferring the graphene layer onto the PDMS surface and are exacerbated during the transfer-printing onto the substrate. As described in the previous section, the defects arise from insufficient wetting of the PDMS surface by water. Since PDMS is hydrophobic and water has high surface tension (≃72 dyn/cm at 23 °C), immediately after a graphene–Au bilayer is scooped by a PDMS stamp, water dewets the PDMS surface in several locations, making the bilayer form contacts to the PDMS surface that is only locally conformal [Supplementary Figs S2(b) and S4(a)]. As the sample is blow-dried using a N_2_ gun, these locally conformal contacts laterally expand, generating narrow wrinkles with water droplets trapped inside, as shown in the right image of Supplementary Fig. S4(a). We speculate that further application of N_2_ pressure causes the water droplets to burst, resulting into defects such as that shown in Supplementary Fig. S3. In fact, as shown in Fig. 2(a), the locations of many defects in the graphene transferred onto the substrate seem to coincide with the intersection of the wrinkles, where relatively large water droplets are expected to form: the linear regions in Fig. 2(a) indicated by the white arrow are where the graphene monolayer is folded, which results from the wrinkles in the graphene–Au bilayer. In contrast, when the ethanol–water mixture was used, its lower surface tension (≃25 dyn/cm at 23 °C^32^) allows a continuous lubrication layer to form between the graphene and PDMS surfaces, providing effective “decoupling” of the bilayer from the PDMS surface. Therefore, no wrinkles, except a few with much smaller heights, were observed in the graphene–Au bilayer on the PDMS stamp [Supplementary Fig. S4(b)]. We found that mild baking at 40 °C removes these wrinkles, resulting into the flat graphene–Au bilayer that is globally conformal to the PDMS stamp, and consequently, successful transfer-printing of the graphene monolayer was achieved without defects, as shown in Fig. 2(b).

The sheet resistance (*R*_sh_) was measured for graphene monolayers transfer-printed on glass substrates, using the van der Pauw method^33^. The size of the graphene monolayers are approximately 1.3 cm by 1.3 cm, and the *R*_sh_ values were obtained with an injected current of 1 mA. In the following, a graphene monolayer transfer-printed onto a final substrate from a PDMS stamp onto which a graphene–Au bilayer was scooped from a bath of water and the ethanol–water mixture are referred to as 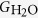 and 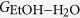, respectively. For 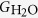, the sheet resistance, averaged over five samples 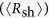 is 3119 Ω/sq, with a minimum equal to 2664 Ω/sq. In contrast, for 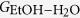, 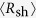 is 914 Ω/sq, with a minimum being 573 Ω/sq. Figure 2(c) shows Raman spectra of graphene monolayers shown in Fig. 2(a,b), where for 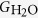 they were obtained from defect-free regions. The spectra show that, for both cases, (i) each Raman peak occurs at the same location (D: 1344 cm^−1^, 2D: 2686 cm^−1^, G: 1588 cm^−1^), (ii) the height of the D peaks is negligible, and (iii) the 2D/G peak ratios are larger than 2.7, confirming that the transfer-printed graphene is indeed a monolayer^34^. This result indicates that significantly larger values of *R*_sh_ for 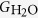, in comparison to that for 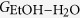, are due not to the properties of graphene in defect-free regions, but to large-scale defects as shown in Fig. 2(a), which has been prevented by decreasing the surface tension in the case of 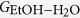. Electrical doping of graphene by ethanol may have occurred for 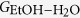 and contributed to the decrease in *R*_sh_, which has been ruled out by an experiment described in Note 1 in Supplementary Material.

Figure 2(d) shows the optical transmission spectra of 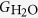 and 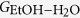 transfer-printed on a 0.7-mm-thick glass substrate, averaged over five samples for each case. Transmittance (*T*), plotted on the *y*-axis, is the intensity of the optical beam transmitted through a glass/graphene sample normalized to that transmitted through a glass substrate. The size of the optical beam at the sample location was approximately 2 mm by 8 mm. For both 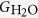 and 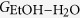, the values of *T* are consistent with what was previously measured for a graphene monolayer on a quartz substrate^12^. The value of *T* for 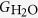 is slightly higher than that for 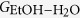, primarily because the absence of graphene in the defects in 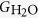 allow more light to be transmitted. Under this hypothesis, the ratio of total area of the defects to the entire area of the graphene sheet (*α*) can be estimated as 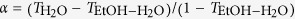, where 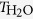 and 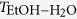 are transmittance of 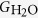 and 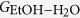, respectively. The value of *α* calculated at each wavelength in Fig. 2(d) ranges from 6% to 8%, which is consistent with our estimation based on optical microscope images.

As expected, successful transfer-printing of graphene requires a defect-free graphene monolayer that is globally conformal to a PDMS stamp. Our proposed technique achieves this with the water–ethanol mixture, which provides a continuous lubrication layer, and with a Au support layer, which allows for its removal without swelling PDMS. Alternatively, one may attempt to obtain a defect-free graphene monolayer on a PDMS stamp by pressing the stamp onto a graphene layer grown on Cu foil and then etching away the Cu foil by floating the Cu/graphene/PDMS on a bath of a Cu etchant. Since the surface of Cu foil commonly used in CVD growth of graphene typically has corrugations on the micron scale^28^, the PDMS attached to the graphene in this case is in contact with the graphene only partially. As a result, subsequent processes such as N_2_ blow-dry and transfer-printing tend to cause defects in the graphene layer, as shown in Supplementary Fig. S5. In fact, it was previously reported that *R*_sh_ of a transfer-printed graphene monolayer by this approach was 4 kΩ/sq, even with a self-release layer inserted for reliable graphene transfer^25^.

**Figure 3:**
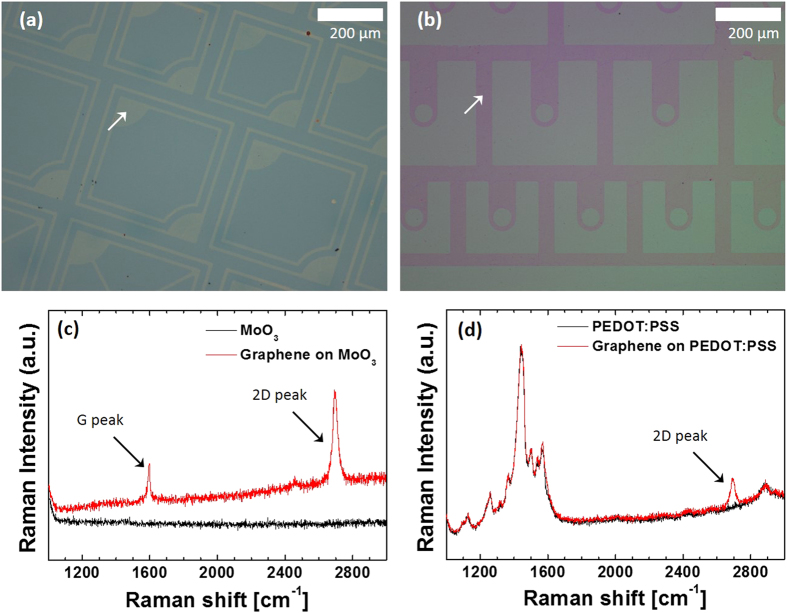
Characterizations of graphene monolayer patterns transfer-printed on materials that can be damaged by a wet process. (**a**,**b**) Optical microscope images of graphene monolayer patterns transfer-printed on (**a**) MoO_3_ and (**b**) PEDOT:PSS. The arrows in (**a**,**b**) indicate regions covered by graphene monolayers. (**c**,**d**) Raman spectra (red) of the patterned graphene on (**c**) MoO_3_ and (**d**) PEDOT:PSS. Also shown are the Raman spectra of the substrates without graphene (black).

To fabricate practical electronic devices where graphene is used as active layers or electrodes, the patterning of graphene is required. Our technique, described in Fig. 1, can achieve this with a simple modification: the process begins with a patterned graphene on Cu foil in Step (a), instead of an unpatterned graphene layer. In our current demonstration, we first prepared a patterned graphene monolayer on Cu foil by etching unpatterned graphene grown on Cu foil by O_2_ RIE using a photoresist etch mask patterned by photolithography. Next, the patterned graphene was transfer-printed on a Si/SiO_2_ substrate coated with MoO_3_ or PEDOT:PSS, both of which are susceptible to degradation when exposed to an aqueous condition or aggressive patterning process. Figure 3(a,b) are optical micrographs of the substrates, where patterned graphene monolayers were transfer-printed in regions indicated by the arrows, showing that the patterns defined on photomasks were replicated in the transfer-printed graphene monolayers. The widths of the smallest features – lines in Fig. 3(a) and arcs in Fig. 3(b) – are 10 *μ*m and 15 *μ*m, respectively, which are identical, within the resolution of the optical imaging system used (~0.5 *μ*m), to those of the corresponding features on the photomask. A closer observation of the pattern edge using a field emission scanning electron microscope (FE-SEM) revealed that it is not straight on the nanoscale, with an “edge resolution” of 50 nm, which is probably attributed to the edge resolution of the photomask patterns and/or limitation of the photolithography process (Supplementary Fig. S7). From this, together with the fact that previously demonstrated transfer-printing-based patterning techniques can create patterns whose size is well below 100 nm^35^, we expect that our technique is capable of creating sub-micrometer graphene patterns, if a nanopatterning process, for example electron-beam^36^, nanoimprint^37^, or nanosphere^38^ lithography, is employed, instead of photolithography. Raman spectra obtained from the graphene transfer-printed on the MoO_3_ show the distinct G and 2D peaks, with the 2D/G intensity ratio of 2.5, and the negligible D peak, suggesting that the quality of the graphene is comparable to that in Fig. 2(b). For the case of the graphene transfer-printed onto the PEDOT:PSS, the peaks associated with graphene, except the 2D peak, cannot be identified due to the overlap with Raman spectra of PEDOT:PSS.

**Figure 4:**
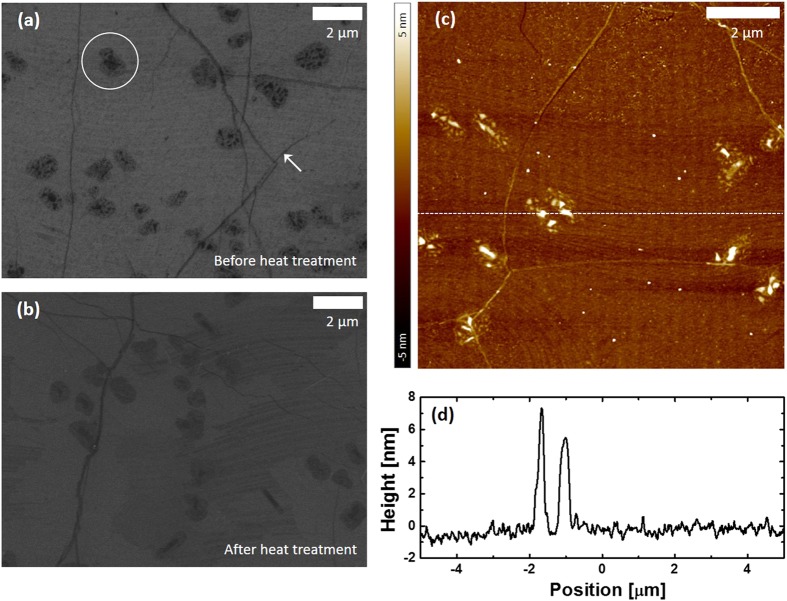
Morphological characterizations of transfer-printed graphene monolayers. (**a**,**b**) Scanning electron microscope images of the graphene surface before (**a**) and after (**b**) annealing at 400 °C under H_2_ and Ar. (**c**) Atomic force microscope image of the sample used in (**a**). (**d**) Height profile of the surface measured along the white dotted line in (**c**).

Next, we observed the surface of the transfer-printed graphene on a Si/SiO_2_ substrate, using a FE-SEM and an atomic force microscope (AFM). As shown in Fig. 4(a), irregularly shaped dark patches, as enclosed by a white circle, are randomly distributed throughout the surface. Also shown are the dark lines, as marked by the white arrow. These two features, patches and lines, are commonly found in the transferred graphene CVD-grown on Cu foil – with the former and latter attributed to graphene multilayers and wrinkles, respectively^28^ – and hence are not caused by our transfer technique. It is also shown that in the patches, there are darker spots with diameters of approximately 150 nm. The surface profile measured using an AFM along the white dotted line in Fig. 4(c) shows that the spots have heights as high as approximately 7 nm [Fig. 4(d)]. To identify the origin of the dark spots, elemental analysis was carried out using a scanning transmission electron microscope capable of energy dispersive x-ray spectroscopy (STEM-EDS). In order to prepare a sample for this analysis, a graphene layer was transfer-printed from a PDMS stamp onto a Si/SiO_2_ substrate coated with a PEDOT:PSS separation layer, and then transferred onto a lacey carbon TEM grid using the conventional wet-transfer method (see the Method Section for the experimental detail). An EDS spectrum obtained from a region shown in Supplementary Fig. S8(a) shows that, in addition to carbon, silicon atoms are present on the graphene surface [Supplementary Fig. S8(b)]. Given many previous reports showing that uncured siloxane oligomers were present on PDMS surfaces^25^,^39^, it is highly likely that the dark spots on the graphene surface are siloxane residues that have been transferred from the PDMS stamp. This speculation was further supported by the fact that the dark spots can be eliminated by annealing the sample at 400 °C under H_2_ and Ar, as shown in Fig. 4(b)^40^. The AFM measurements [Fig. 4(c,d)] show that the surface in the background, that is, regions away from the patches and lines, is much rougher than that of a clean graphene surface^41^, suggesting that the oligomer residues are also present throughout the surface, not only on the multilayer regions.

The morphological and elemental characterizations of the surface of transfer-printed graphene discussed above help determine the range of application of our technique in its current form. Since the oligomer residues are likely to be present only on the top surface, that is, the graphene surface that used to be in contact with the PDMS, our technique can be applied to fabrication of (i) devices where only the bottom surface of the graphene electrode is involved in injection or collection of charge carriers, such as LEDs and solar cells, made of organic semiconductors^42^ or organometal trihalide perovskite compounds^43^, with top graphene electrodes, and (ii) devices whose graphene electrodes are used to establish electric fields without charge carrier transport, such as thin-film transistors with graphene gate electrodes^44^ and terahertz wave modulators^13^,^14^,^15^. Meanwhile, when charge carrier injection or collection occurs in both sides of the graphene layer, such as in tandem LEDs and solar cells where it is part of the interlayers, our technique is not applicable. Therefore, expanding the range of application of our technique by eliminating the oligomer contamination, possibly with the following modification, is important future work: replacing PDMS with other stamp material that can be completely cured; or depositing a blocking layer on the PDMS surface to prevent possible transfer of uncured oligomers onto the graphene surface, with a potential candidate being a pressure sensitive adhesive layer^45^,^46^.

A possible concern with choosing Au as a material for the support layer is its high cost. Although Au is an expensive material, the thinness of the support layer translates to a small amount of Au used per area: the 200-nm-thick Au support layer corresponds to 0.386 mg of Au per cm^2^, whose cost may not be of a concern for many device applications. Another concern may be effects of the Au etchant on the electrical quality of transfer-printed graphene. In our experiments, exposure of graphene to the Au etchant is rather brief (<5 min), after which samples are thoroughly rinsed with clean water. As a result, the elemental characterization of the transfer-printed graphene layer in Fig. S8 does not show any peaks associated with the Au etchant, such as those of iodine. Furthermore, through a simple experiment described in Note 2 in Supplementary Material, we have confirmed that the change in *R*_sh_ due to the Au etchant is negligible. Finally, we note that Au in our demonstration can be considered as a representative material for the support layer satisfying the following conditions — it must not be etched away by the Cu etchant, and can be removed without swelling PDMS and damaging graphene — and hence can be replaced by other material, not necessarily metallic, that is more cost-effective and/or easily removed.

## Conclusion

In summary, we have developed a low temperature, dry process capable of transfer-printing a patterned graphene monolayer grown on Cu foil on a target substrate. Two features distinct from the conventional wet-transfer method^18^ – the use of a support layer composed of Au, instead of PMMA, and the decrease in surface tension of the liquid bath on which a graphene–Au bilayer floats – allow one to obtain a graphene monolayer on a PDMS stamp without defects that would otherwise arise. Subsequently, the graphene is transfer-printed from the stamp onto a target substrate. The characteristics of a graphene monolayer transfer-printed using our technique are comparable to those obtained with the conventional wet-transfer method, with a sheet resistance as low as 573 Ω/sq and optical transmittance of 97.4% at 550 nm. In addition, with pre-transfer patterning of graphene on Cu foil using conventional patterning processes, our technique is capable of creating graphene monolayer patterns on materials that are easily degraded when exposed to high-temperature processes, organic solvents, or aqueous chemicals. As an example, using photolithography followed by reactive-ion etch to pattern graphene monolayers on Cu foil and then transfer-printing them, we have obtained graphene monolayer patterns on MoO_3_ and PEDOT:PSS, with the smallest feature size and edge resolution of ≃10 *μ*m and 50 nm, respectively. Immediate application areas of this technique include organic electronic devices whose top electrodes are composed of graphene. Moreover, by eliminating siloxane oligomer residues on graphene using alternate stamp material, the technique can be further applied to devices whose graphene electrodes are in their interiors, such as tandem LEDs and solar cells. Finally, with possible appropriate modification, it may also be applied to dry-transfer of other two-dimensional materials, including boron nitride^47^ and molybdenum disulfide^48^.

## Methods

### CVD-grown graphene monolayer

A graphene monolayer on Cu foil was grown in a CVD system consisting of a tubular quartz reactor and a furnace. Experimental details described in ref.^12^ were closely followed except the following: Cu foil was annealed under a 5 sccm flow of H_2_ at 20 mTorr, and during growth, the reactor was filled with a mixture of CH_4_ and H_2_ at a total pressure of 150 mTorr, whose flow rates are 35 and 5 sccm, respectively.

### Low-temperature, dry transfer-printing process

Low-temperature, dry transfer of graphene monolayers was carried out by following processes described in Fig. 1. To form a support layer on a graphene monolayer on Cu foil, a 200-nm-thick Au layer was deposited by thermal evaporation in high vacuum (1 Å/s, ~10^−7^ Torr) (b). To etch away the Cu foil, the Cu/graphene/Au multilayer was floated on an ammonium persulfate solution, prepared by dissolving 10 g of ammonium persulfate (Sigma Aldrich) in 500 ml of water (c). After the etch was completed, the graphene–Au bilayer was scooped with a glass slide, and then transferred on a bath of water to remove residual ammonium persulfate. Next, the graphene–Au bilayer was moved onto a bath composed of an ethanol–water mixture (70 vol % ethanol and 30 vol % water), from which the bilayer was scooped by and transferred onto a PDMS stamp (d). The sample was then blow-dried using a N_2_ gun (e), and was further dried on a hot plate at 40 °C for more than 4 h. The Au support layer was etched using an ammonium iodide solution (LAE-202, Cowon Innotech. Inc.) (f), after which the PDMS/graphene sample was rinsed with water. After water droplets on the sample were blown away using a N_2_ gun, the graphene-coated PDMS stamp was gently pressed onto a target substrate, inducing intimate contact throughout the substrate area (g). Before separation of the stamp from the substrate, the sample was stored at room temperature for 1 h under a pressure of 9.9 kPa, and then placed on a hot plate at 70 °C for 10 min without application of pressure. Finally, the stamp was carefully peeled off from the substrate (h), resulting in the transfer-printed graphene monolayer on the target substrate.

### Transfer-printing of patterned graphene layers

In this process, a graphene monolayer on Cu foil was first patterned using conventional photolithography and reactive-ion etch, as described in Supplementary Fig. S6. A 1.5-*μ*m-thick photoresist (AZ GXR-601, 14cP) was spin-coated on a Cu/graphene sample, and then patterned by photolithography. The patterned graphene on Cu foil was obtained, when the graphene in the areas not covered by the photoresist was etched by reactive ion etch in O_2_ (100 W, 0.1 Torr, 20 s, 50 sccm). Performing the processes described in Fig. 1 with this sample, instead of unpatterned graphene on Cu foil, we transfer-printed a patterned graphene monolayer on a target substrate coated with a 75-nm-thick PEDOT:PSS or a 20-nm MoO_3_ layer. The target substrate was a 500-*μ*m-thick Si substrate pre-coated with a 285-nm-thick thermal SiO_2_ layer, and the PEDOT:PSS (Heraeus) and MoO_3_ (LTS Chemical Inc.) layers were deposited by spin-coating (3000 rpm, 30 s) and thermal evaporation in high vacuum (1 Å/s, ~10^−7^ Torr), respectively.

### Sample preparation for the elemental analysis

Samples for the elemental analysis were prepared following the processes described in Supplementary Fig. S9. After a graphene monolayer was transfer-printed from a PDMS stamp onto a PEPOT:PSS layer using our transfer method (a), a layer of PMMA was deposited on the graphene layer by spin coating at 3000 rpm for 30 s (b). The PMMA solution was prepared by dissolving PMMA (138 mg, Sigma Aldrich) into chlorobenzene (3 mL, Sigma Aldrich). The sample was then immersed into a water bath, separating the PMMA–graphene bilayer from the Si/SiO_2_ substrate as the PEDOT:PSS layer was dissolved (c). Next, the bilayer was transferred to another water bath and kept floating on it for more than 24 h to ensure that PEDOT:PSS remaining on the graphene surface was removed. Then, the bilayer was scooped with a lacey carbon TEM grid (Ted Pella, Inc.) (d), after which the grid was placed on a hot plate at 40 °C for more than 2 h. Finally, the PMMA layer was removed by acetone (e), resulting in the graphene monolayer on the TEM grid (f).

### Characterization of transfer-printed graphene layers

The surface morphology of transfer-printed graphene layers was characterized using a FE-SEM (JSM-6700F, JEOL) and an AFM (Dimension Edge, Bruker). The sheet resistance was measured using a source meter (2400, Keithley) and a multimeter (34410A, Agilent). The Raman spectra were obtained using a confocal Raman microscope (inVia, Renishaw) with an excitation wavelength of 514.5 nm emitted from an Ar laser. An ultraviolet–visible spectrophotometer (Lambda 35, Perkin Elmer) was used to measure the optical transmittance spectra. The elemental analysis was carried out using a STEM equipped with an EDS (JEM-2100F, JEOL).

## Additional Information

**How to cite this article**: Cha, S. *et al*. Low-Temperature, Dry Transfer-Printing of a Patterned Graphene Monolayer. *Sci. Rep*. **5**, 17877; doi: 10.1038/srep17877 (2015).

## Supplementary Material

Supplementary Information
