# Supplementary material for: Low-Temperature, Dry Transfer-Printing of a Patterned Graphene Monolayer
Source: Sci Rep. 2015 Dec 9;5:17877. doi: 10.1038/srep17877 (PMC4673461; doi:10.1038/srep17877)
Supplement: Supplementary Information [file srep17877-s1.pdf]

Supplementary Information

Low-Temperature, Dry Transfer-Printing of a Patterned Graphene Monolayer

Sugkyun Cha,[1,*] Minjeong Cha,[1,*] Seojun Lee,[1] Jin Hyoun Kang,[2] and Changsoon Kim[1,3,†]

[1] *Program in Nano Science and Technology, Graduate School of Convergence Science and Technology, Seoul National University, Seoul 151-742, Republic of Korea*

[2] *Department of Chemistry, Seoul National University, Seoul 151-747, Republic of Korea*

[3] *Advanced Institutes of Convergence Technology, Suwon, Gyeonggi 443-270, Republic of Korea*

[*] These authors contributed equally.

[†] changsoon@snu.ac.kr

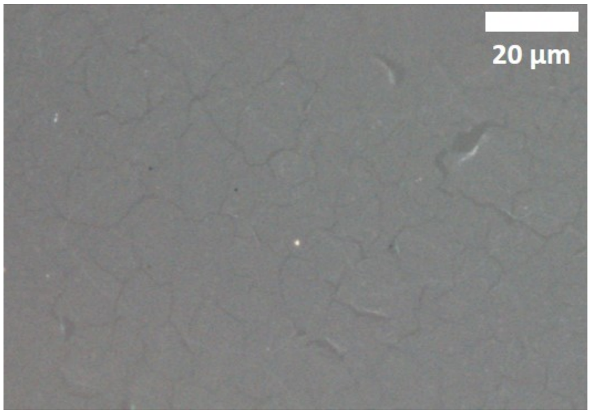

Figure S1. Optical microscope image of a graphene monolayer transferred onto a PDMS stamp via the conventional wet-transfer method that uses a PMMA support layer.

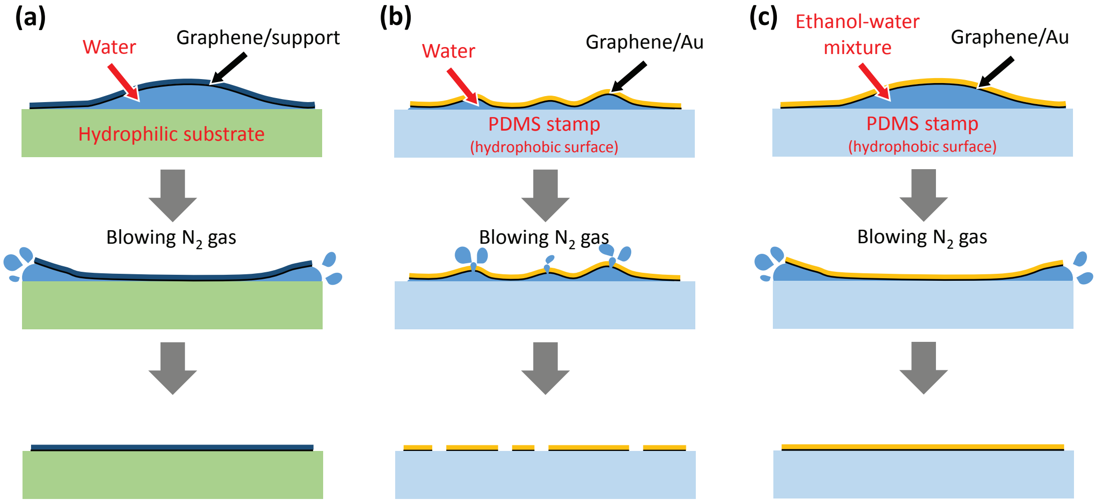

Figure S2. **Importance of wetting of a substrate in determining the quality of a graphene–support bilayer on the substrate: schematic illustration.** (a) In the conventional wet-transfer case, sufficient wetting of a hydrophilic substrate by water leads to conformal contact between the graphene and the substrate without wrinkles when the sample is blow-dried using N_2 gas. (b) When a PDMS stamp, whose surface is hydrophobic, is used instead of a hydrophilic substrate, water does not form a continuous layer between the graphene and the PDMS stamp. Consequently, a blow-drying process in this case causes water droplets trapped between the graphene and the PDMS to burst, damaging the graphene–Au bilayer. (c) In our method, the surface tension of the bath is decreased using a mixture of water and ethanol, which sufficiently wets the PDMS surface. As a result, the graphene–Au bilayer whose quality is comparable to that in the conventional wet-transfer is obtained on the PDMS stamp.

Note 1. Effect of ethanol. To quantify the possible contribution of ethanol in decreasing the sheet resistance (R_{sh}) of $G_{\mathrm{EtOH-H_2O}}$, we performed the following experiment:

(i) A graphene layer was transfer-printed onto a Si substrate coated with 300-nm-thick SiO_2 layer using our method.

(ii) The sample was annealed at 400 °C for 3 hr under H_2 and Ar at a total pressure of 90 mTorr, which eliminates ethanol molecules adhered to the graphene.

(iii) the sample was exposed to ethanol for 5 min, and subsequently dried using a N_2 gun and annealed at 40 °C for 4 hr.

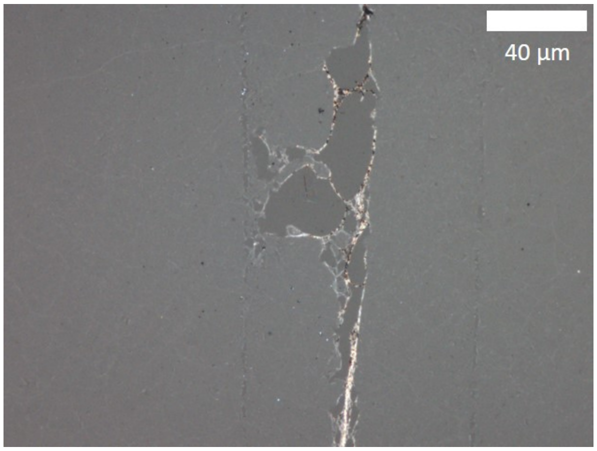

Figure S3. Optical microscope image of a graphene layer transferred on a PDMS stamp using a water bath as in Step (d) in Fig. 1.

The sheet resistance values measured before and after Step (iii) using the van der Pauw method are 1334 and 1144 Ω/sq, respectively. Although the sheet resistance was slightly decreased due to exposure to ethanol, the magnitude of change is too small to explain the significant difference in R_{sh} between $G_{\mathrm{EtOH–H_2O}}$ and $G_{\mathrm{H_2O}}$.

Note 2. Effect of Au etchant. To demonstrate whether the Au etchant influences the quality of graphene or not, we performed the following experiment:

(i) A graphene layer was transfer-printed onto a Si substrate coated with 300-nm-thick SiO_2 layer using our method.

(ii) The sample was annealed at 400 °C for 3 hr under H_2 and Ar at a total pressure of 90 mTorr, which eliminates possible dopants arising from the Au etchant.

(iii) The sample was exposed to the Au etchant for 5 min, followed by repeated thorough rinsing with clean water.

The R_{sh} values measured before and after Step (iii) using the van der Pauw method are very similar to each other, 770 and 822 /sq, respectively.

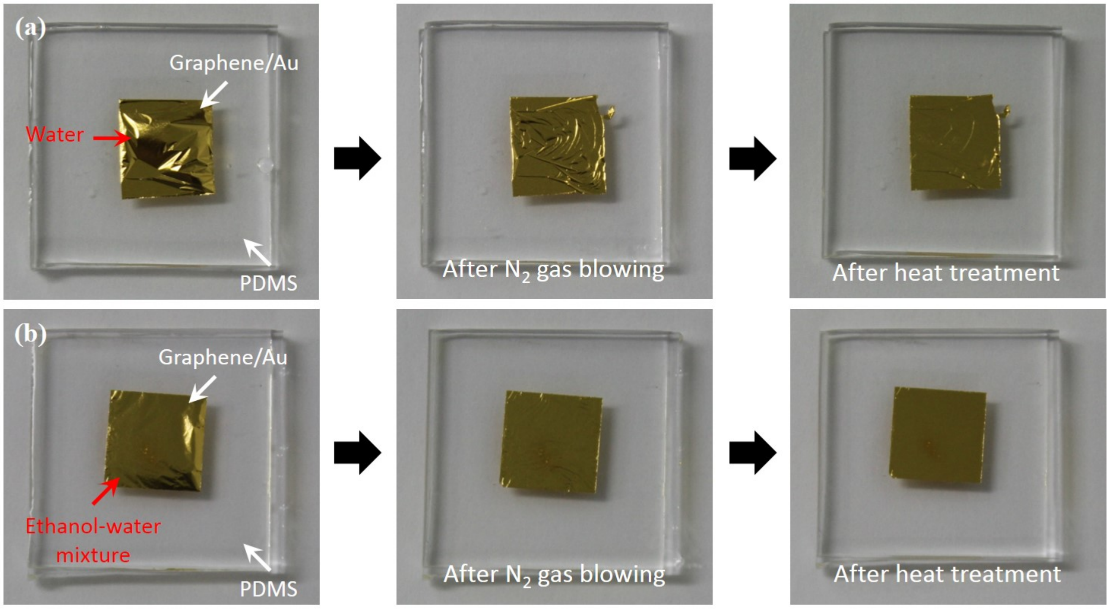

Figure S4. **Importance of wetting of a substrate in determining the quality of a graphene–Au bilayer on the substrate: digital images.** These are digital images of a graphene–Au bilayer scooped up from a bath with a PDMS stamp, as shown in Fig. 1(d). (a) When a water bath is used, water dewets the PDMS surface in several locations before a blow-dry process (left). After blow-drying the sample with a N_2 gun, the bilayer has many wrinkles, in which water is trapped (center). Although mild annealing on a hot plate at 40 °C for 4 h decreases the heights and number of the wrinkles as it removes the residual water, the wrinkles cannot be completely eliminated (right). (b) In contrast, when the bilayer was scooped up from a mixture of water and ethanol, the mixture liquid forms a continuous lubrication layer between the bilayer and the PDMS stamp throughout the surface (left). As a result, N_2 flow aiming at the center of the bilayer displaces the liquid outward, resulting in conformal contact between the bilayer and the PDMS, almost throughout the surface (center). Small number of wrinkles with smaller heights than those in (a) can be removed after heat treatment on a hot plate at 40 °C for 4 h (right).

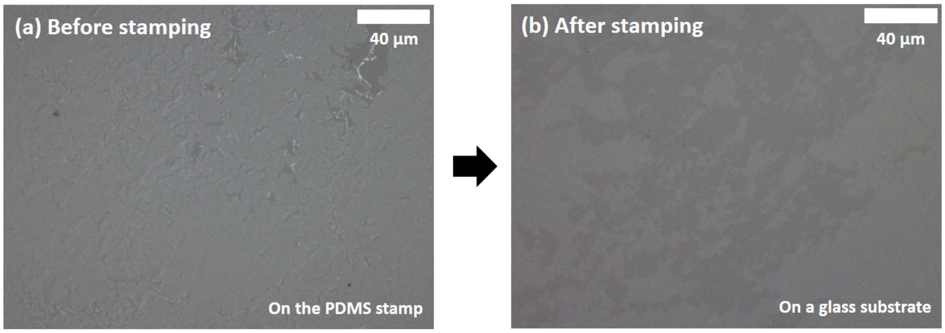

Figure S5. (a) Optical microscope image of a PDMS stamp coated with a graphene layer, showing defects in the graphene. Here, the graphene layer was deposited on the PDMS by pressing the stamp onto the graphene layer on Cu foil, and floating the resulting Cu/graphene/PDMS structure on a Cu etchant solution to etch the Cu layer. (b) Optical microscope image of the graphene layer transfer-printed on a glass substrate from a stamp such as that shown in (a), showing a large number of defects.

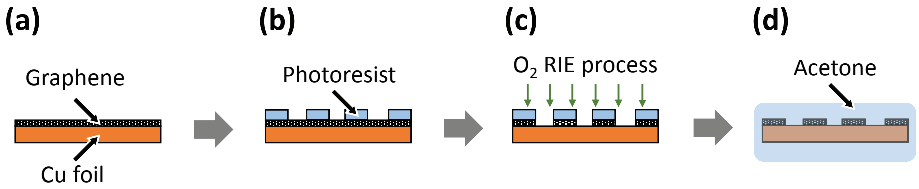

Figure S6. **Pre-transfer patterning of graphene layers.** (a) CVD-grown graphene layer on Cu foil. (b) A photoresist layer on the Cu/graphene patterned by conventional photolithography. (c) Reactive-ion etch of the graphene. (d) Removal of the photoresist by acetone.

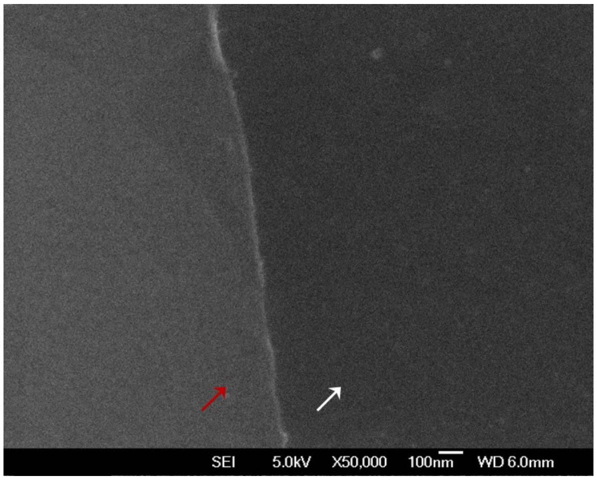

Figure S7. SEM image of a graphene layer transfer-printed on MoO_3, showing a pattern edge resolution of approximately 50 nm. The regions indicated by red and white arrows are the graphene and MoO_3 surfaces, respectively.

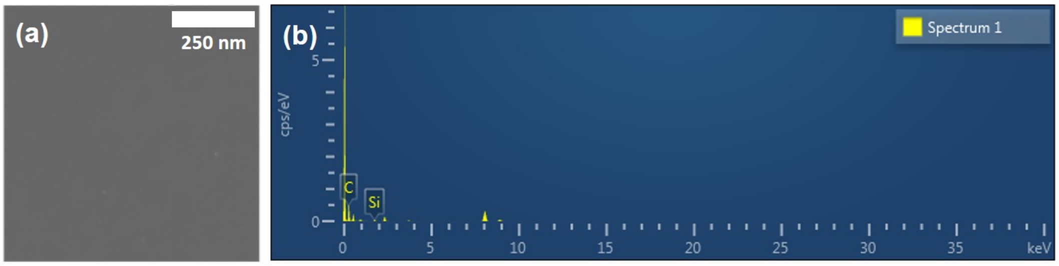

Figure S8. **Elemental characterization of a transfer-printed graphene layer.** (a) Scanning transmission electron microscope image of the region where an energy dispersive x-ray spectroscopy (EDS) was performed. (b) EDS spectrum obtained from the region shown in (a).

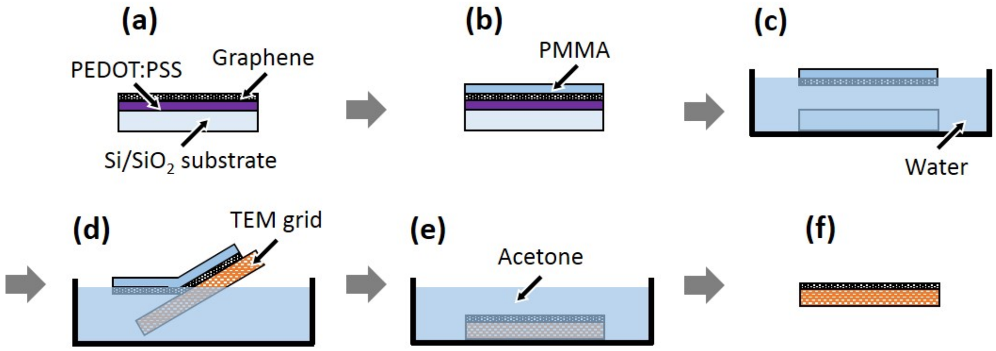

Figure S9. **Sample preparation for elemental analysis.** (a) Transfer-printing of a graphene layer using the technique proposed in this paper onto a Si/SiO_2/PEDOT:PSS. (b) Deposition of a PMMA layer by spin coating. (c) Lifting off the graphene–PMMA bilayer by dissolving the PEDOT:PSS layer in water. (d) Transferring the graphene–PMMA bilayer onto a lacey carbon grid. (e) Removal of the PMMA layer using acetone. (f) The graphene layer transfer-printed onto the target substrate is now placed on the lacey carbon grid for the elemental analysis.
